# Cardiomyopathies: Classification, Clinical Characterization, and Functional Phenotypes

**DOI:** 10.1155/2012/870942

**Published:** 2012-12-04

**Authors:** Danuta Szczesna-Cordary, Sachio Morimoto, Aldrin V. Gomes, Jeffrey R. Moore

**Affiliations:** ^1^Department of Molecular and Cellular Pharmacology, University of Miami Miller School of Medicine, Miami, FL 33136, USA; ^2^Department of Clinical Pharmacology, Kyushu University Graduate School of Medicine, Fukuoka 8128582, Japan; ^3^Department of Neurobiology, Physiology and Behavior, University of California, Davis, CA 95616, USA; ^4^Department of Physiology and Biophysics, Boston University School of Medicine, Boston, MA 02118, USA

Cardiomyopathy is a category of disorders that affect the cardiac muscle and can cause extensive disability in a large and ethnically diverse population. It has been classified by the World Health Organization into three main types, based on anatomical and physiological features: dilated (DCM), hypertrophic (HCM), and restrictive (RCM). While DCM and RCM are associated with a progressive disease phenotype, heart failure, and sudden death, HCM is the leading cause of sudden cardiac death (SCD) in young athletes and is one of the most common forms of heart diseases affecting children. One review article in this issue by C. McCartan et al. discusses how the traditional classification of cardiomyopathies based on morphology has evolved due to rapid advances in our understanding of the genetic and molecular bases for many of these clinical entities. The implications of genetic testing have been discussed as being extremely valuable in the prognosis and treatment of cardiomyopathies. The authors emphasize that such testing may allow clinicians to move beyond unexplained ventricular abnormalities, identify positive patients and their genetic cause, and predict likely outcomes. A similar need for genetic testing has been voiced in another review article in this issue, by R. Parvari and A. Levitas, which addresses the pathogenesis of hereditary DCM. The authors strongly argue that the idea of early identification of the disease-causing mutations as presymptomatic interventions in DCM has proven valuable in preventing morbidity and mortality. DCM may be either sporadic or familial. A diagnosis of familial DCM is assigned when it occurs in at least two closely related family members or there was an occurrence of SCD at a young age. The genetic factor contributing to the manifestation of the disease can be classified either as a disease-causing gene mutation, as the etiology of monogenic disease, or as a disease-associated gene polymorphism that is involved in the pathogenesis of a multifactorial disease. 

To date, hundreds of mutations in genes encoding all major sarcomeric proteins have been identified to cause HCM, DCM, or RCM. These include proteins essential for force production (*β*-cardiac myosin and actin), regulation of cardiac muscle contraction (tropomyosin and troponin), and proteins responsible for maintaining stability of the sarcomere (myosin-binding protein C, titin). The vast majority of cardiomyopathy mutations have been found in MYH7 gene encoding the *β*-myosin heavy chain (MHC). Mutations in myosin light chains MYL2 and MYL3 encoding the regulatory and essential light chains, respectively, are relatively rare, but they are also associated with malignant outcomes in young individuals. In fact, most of myosin essential light chain (ELC) mutations have been associated with SCD. In one of the articles of this issue, P. S. Andersen et al. identified a novel MYL3 mutation, V79I (Valine→Isoleucine), in a 38-year-old HCM patient with no visible cardiac symptoms. Mutation-positive family members were also asymptomatic, and no information was available on previous occurrences of SCD in two prior generations. At the molecular level, the mutation was predicted to disrupt the interaction of ELC with the MHC, but future biochemical studies are necessary to reveal the possible mechanism of this V79I-induced HCM. In another paper by S. K. Gollapudi and M. Chandra, two cardiac troponin C (TnC) mutations were studied: an HCM-related, L29Q (Leucine→Glutamine), and a DCM-related, G159D (Glycine→Aspartic acid) mutation. Using mutant reconstituted skinned rat cardiac papillary muscle fibers, the authors provided new evidence for the mechanism by which these two TnC mutations bring about global change in myofilament mechanodynamics. The L29Q mutation caused a small decrease in myofilament Ca^2+^ sensitivity, which was linked to an increase in the tropomyosin-troponin regulatory unit “off” rate. The G159D mutation resulted in an increase in the cross-bridge cycling rates, “f” and “g”, with no effects on maximal tension and Ca^2+^ sensitivity. These different molecular effects were predicted to result in two different disease phenotypes in humans. The L29Q-induced HCM phenotype was proposed to evolve due to a compensatory response of the myocardium to decreased tension caused by an attenuation of myofilament Ca^2+^ sensitivity. The G159D-mediated DCM phenotype was hypothesized to be a result of increased cost of tension maintenance, subjecting the heart to a chronic stress that ultimately could lead to ventricular dilatation. 

Heart failure is the common end-stage condition of various cardiovascular disorders including cardiomyopathies and is characterized by a progressive decrease in cardiac output combined with insufficient or absent compensatory mechanisms. The interplay between heart development and disease has been the subject of intense research with the characterization of the gene regulatory networks involved in the development of cardiac dysfunction. Another paper in this issue, by A. T. Mikhailov and M. Torrado, focuses on the role of myocardin and myocardin-related transcription factors (MRTF) in heart failure and cardiac hypertrophy. From the combined knowledge of the molecular and functional domains of the molecules as well as the phenotypes of several knockout models, it was concluded that myocardin and MRTF play a central role in cardiac formation, function, and disease and present themselves as potential candidates as therapeutic targets for the treatment of heart failure. Gene-manipulated mouse models that closely recapitulate the clinical phenotypes of human inherited cardiomyopathies are invaluable in demonstrating the true cause of the disease and exploring pathogenic mechanism and effective drugs. Ultrasound imaging has been increasingly applied to identify and characterize structural and functional features of different cardiac phenotypes in mouse models of cardiac disease. The laboratory of Dr. X. P. Huang from Florida Atlantic University is among the first to assess cardiac function using a high-resolution echocardiography on transgenic mice with various cardiomyopathies. In their review article, G. Chen et al. discuss several important technical developments in the conventional echocardiography, pulsed Doppler, and tissue Doppler imaging for assessment of cardiac morphology and function in mouse models of human inherited cardiomyopathies. 

In summary, this special issue discusses several aspects of cardiomyopathies related to mutations in myofilament proteins. These papers and reviews as well as our own research using animal models of human heart disease suggest that finding the mutation-specific molecular causes of HCM, DCM, or RCM is absolutely necessary in the development of target-specific rescue strategies to ameliorate or reverse the effects of inherited cardiomyopathies. The classification and clinical characterization of cardiomyopathies will continue to evolve as we unravel these molecular mechanisms by which HCM, DCM, or RCM-associated mutations lead to heart disease and SCD.

*Danuta Szczesna-Cordary* *Sachio Morimoto* *Aldrin V. Gomes* *Jeffrey R. Moore*

